# iGepros: an integrated gene and protein annotation server for biological nature exploration

**DOI:** 10.1186/1471-2105-12-S14-S6

**Published:** 2011-12-14

**Authors:** Guangyong Zheng, Haibo Wang, Chaochun Wei, Yixue Li

**Affiliations:** 1 Key Laboratory of Systems Biology, Shanghai Institutes for Biological Sciences, Chinese Academy of Sciences, 320 Yueyang Road, Shanghai 200031, China; 2 Shanghai Center for Bioinformation Technology, 100 Qinzhou Road, Shanghai 200235, China; 3 School of Life Sciences and Biotechnology, Shanghai Jiao Tong University, 800 Dongchuan Road, Shanghai 200240, China; 4 Institute of Biochemistry and Cell Biology, Shanghai Institutes for Biological Sciences, Chinese Academy of Sciences, 320 Yueyang Road, Shanghai 200031, China

## Abstract

**Background:**

In the post-genomic era, transcriptomics and proteomics provide important information to understand the genomes. With fast development of high-throughput technology, more and more transcriptomics and proteomics data are generated at an unprecedented rate. Therefore, requirement of software to annotate those omics data and explore their biological nature arises. In the past decade, some pioneer works were presented to address this issue, but limitations still exist. Fox example, some of these tools offer command line only, which is not suitable for those users with little or no experience in programming. Besides, some tools don’t support large scale gene and protein analysis.

**Results:**

To overcome these limitations, an integrated gene and protein annotation server named iGepros has been developed. The server provides user-friendly interfaces and detailed on-line examples, so most researchers even those with little or no programming experience can use it smoothly. Moreover, the server provides many functionalities to compare transcriptomics and proteomics data. Especially, the server is constructed under a model-view-control framework, which makes it easy to incorporate more functions to the server in the future.

**Conclusions:**

In this paper, we present a server with powerful capability not only for gene and protein functional annotation, but also for transcriptomics and proteomics data comparison. Researchers can survey biological characters behind gene and protein datasets and accelerate their investigation of transcriptome and proteome by applying the server. The server is publicly available at http://www.biosino.org/iGepros/.

## Background

In the post-genomic era, one of the important goals of biological research is to explain genome contexts and understand the function of genetic information [1]. Experiments on transcription and translation levels are widely carried out to decipher the functions behind a genomic sequence. In recent decades, some high-throughput technologies, such as microarray, next generation sequencing (NGS), and mass spectrometry (MS), have been introduced to meet these requirements. For example, microarray technology is often used to detect mRNA expression under a specific physiological condition [2,3]. More recently, RNA-seq technology was developed to inspect RNA expressions on the whole genome scale [4,5]. While for the mass spectrometry technology, it is generally followed with liquid chromatography method to quantify protein expression levels [6,7].

After a large-scale gene or protein set is obtained by preliminary analyzing the raw data of transcriptomics and proteomics experiments, annotating those candidate genes and proteins will be executed to survey their biologic characters. During this annotation process, collecting mappings of genes and proteins to gene ontology (GO) is regarded as the primary step, which can help people decipher their roles in biological process, cellular component, or molecular function aspect. Many GO annotation tools have been developed to browse, search, and visit GO terms for genes and proteins. For example, Carbon and his colleagues provided a website tool for online accessing GO terms [8]. The Bioconductor community released some GO packages written in R language, which provided a comprehensive annotation method for gene and protein sets [9,10]. Besides, for a large size gene or protein set, an enrichment analysis is necessary for finding out whether the set shows statistical significance on some GO terms [11]. In recent years, some pioneer works have been reported for this purpose [12-14]. In addition, the KEGG pathway is regarded as another pivotal term for annotating the functions of genes and proteins. Pathway information can help people understand relations between genes and proteins as well. In 2007, Moriya and his colleagues offered a KEGG pathway annotation server for high-throughput data, which can automatically generate pathways based on annotation results of the input data [15]. Furthermore, an enrichment analysis on KEGG pathways is required for deeply investigating the relationships between genes and proteins. For example, a knowledge-base website named DAVID has been created for GO and pathway annotation [16]. These pioneer works mentioned above provide useful GO and pathway annotation tools for the biologist community, but some limitations are still exist, which prevents people to analysis large-size data sets produced by high-throughput technology. First, some of these tools only offer a command line mode for users, which is not convenient for users with little or non programming skill. Secondly, some tools do not support large data set analysis on one time. Therefore, they are not suitable for annotation of datasets generated by a high-throughput technology. At last, experiments executed on both transcription and translation levels need integration software, which can annotate gene and protein sets simultaneously and combine the annotation results. To our knowledge, there is no available software for this purpose.

In this paper, we present an integrated web server with a user friendly interface for gene and protein annotation. The server supports large size datasets as inputs, so it can be used as an analysis tool for high-throughput experiments. Especially, it offers a powerful association module to combine results of gene and protein annotations. This can help people effectively analyze datasets produced by assays launched on both transcriptional and translational aspects. In a word, the server can help to explore the biological nature behind gene and protein data.

## Methods

**Figure 1:**
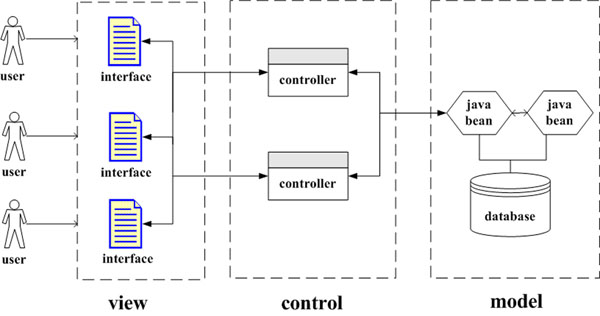
Framework of iGepros based on MVC architecture

The server was constructed under a Model-View-Control (MVC) architecture, which was widely adopted in the IT community [17]. Detailed information of the architecture was shown in figure 1. The MVC architecture is a flexible framework, which separates the graphic interface, the control module, and the business logic. In this framework, the model and view section denote business logic and graphic interface respectively, while the third section of the framework indicates control module. Since three sections of the framework are partitioned, each section can be implemented independently, which is considered as an outstanding merit of this architecture. In this work, the Java Server Pages (JSP) technology was used to implement the interface, the view section of the architecture, which collected requirements from users and returned outcomes to them. The Java Server Applet (servlet) technology was adopted to establish a controller, which received information from the view section and allocated tasks to appropriate model sections. Meanwhile, the Java Bean technology was utilized to build up the model sections. For data storage, a MySQL database (version 5.0.45) was used as the repository. In addition, Apache Tomcat (version 5.5.23) was employed as the servlet engine to support the web interface. Finally, the server was deployed on a Linux system.

In practice, two data uploading methods were developed for large data set analysis. On one hand, users can directly copy and paste their data with a valid format in the textbox offered by the server. On the other hand, they can choose a local data file with a proper format and upload it to the server. Then, controllers of the server called relative program to finish users’ tasks. Subsequently, results of those tasks were returned with a legible table manner, so that users could quickly read and save outputs. In this work, the server was deployed on a cluster machine with 4 CPUs and 8GB memory so as to ensure its performance for large size data sets. Currently, the server can handle a gene or protein list contained 300 database IDs on one time, and it can be done within 20 minutes.

## Results

iGepros is a user-friendly web server aiming to provide powerful annotation tools for large size gene and protein sets. Furthermore, the server offers some useful association tools to connect gene and protein annotation results. It can combine outcomes of transcriptomics and proteomics experiments, which help researchers to understand the biological nature in transcriptional and translational levels. In order to make researchers analyze massive data conveniently, three modules (named Gene module, Protein module, and Gepro module) are set up. Detailed functionality of these three modules is described in followed sections.

### Gene module

**Figure 2:**
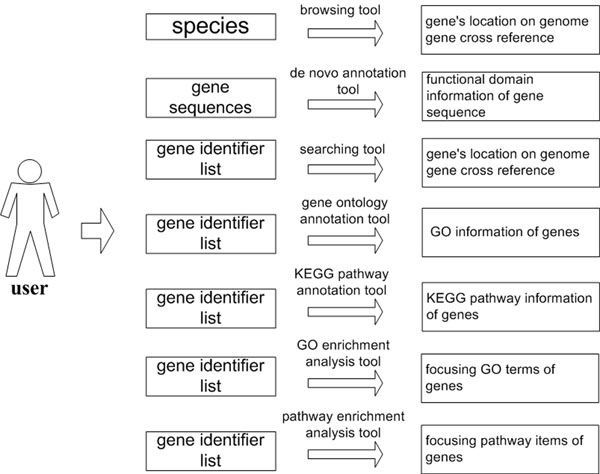
Data flow of the Gene module

The module was designed to meet the requirement of interpreting gene sets. Currently, following tools are included in the module: (1) A gene browsing tool, which provides gene’s location on a genome and its cross reference index with a table format. In this tool, users can select an interested model organism (Human, Mouse, Rat, Chick, Cow, Zebrafish, Arabidopsis), then gene information of the species will be returned. Currently, following gene information are offered: chromosome, strand, start position, end position, chromosomal location, gene name, Entrez gene ID, Unigene ID, Refseq ID and Ensemble ID. (2) A gene searching tool, which is an agile tool designed for gene cross reference query. Currently, the tool supports following query terms: gene name, Entrez gene ID, Unigene ID, Refseq ID, and Ensemble ID. (3) A *de novo* annotation tool for gene sequence, which is an annotation tool for gene sequence with no functional information. For a gene sequence, when it is submitted to the server, it will be translated into amino acid sequences with 6 frames. Then the amino acid sequence with maximum length open reading frame is delivered to the “InterProScan” software for functional domain scanning. Finally, users can understand biological roles of the gene sequence through inspecting results of functional domain scanning. (4) A gene annotation tool for GO terms, which is an annotation tool used for GO information collection. In this tool, it accepts gene identifiers (gene name, Entrez gene ID, Unigene ID, Refseq ID, Ensemble ID) as inputs and returns GO terms of genes as outputs. (5) A gene annotation tool for KEGG pathway, which is utilized to collect pathway information for genes. In this tool, it receives gene identifiers (gene name, Entrez gene ID, Unigene ID, Refseq ID, and Ensemble ID) first, and then gives pathways information that these genes involved. (6) A GO enrichment analysis tool for genes, which is a statistical tool used to find out the common GO terms for interested genes. In this tool, it receives a gene list first, and then carries out enrichment analysis via a hypergeometric distribution with 0.05 as P-value threshold. (7) A pathway enrichment analysis tool for genes, similar to GO enrichment analysis tool, which is developed to give the common pathways for genes through estimation of hypergeometric distribution. Data flow of the Gene module was shown in Figure 2.

### Protein module

**Figure 3:**
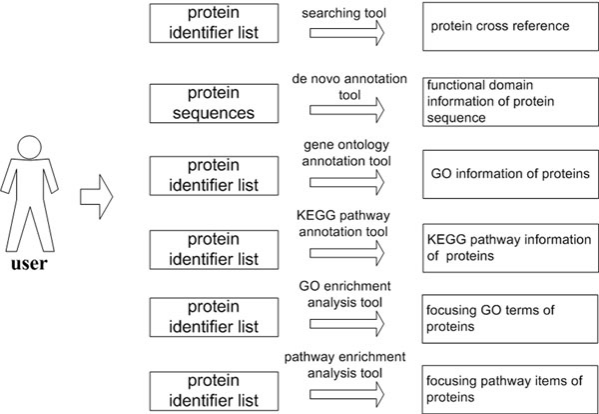
Data flow of the Protein module

This module was designed to meet analysis requirement of protein sets. Similar to the gene module, some tools about protein annotation and enrichment analysis are included in the module: (1) A protein searching tool, which is a query tool built for protein cross reference search. Currently, it supports 3 types of protein identifiers: the accession number of IPI database, accession number of Swiss-Prot database, and Refseq ID. (2) A *de novo* annotation tool for protein sequence, which is an annotation tool for protein sequence with no functional information. (3) A protein annotation tool for GO terms. (4) A protein annotation tool for KEGG pathway. The above two tools are established aiming to giving proteins’ mappings to GO terms and pathway information. (5) A GO enrichment analysis tool for proteins, and (6) A pathway enrichment analysis tool for proteins. These two above tools are developed to find out GO terms and pathway items for interested proteins respectively. Data flow of the Protein module was shown in figure 3.

### GePro module

**Figure 4:**
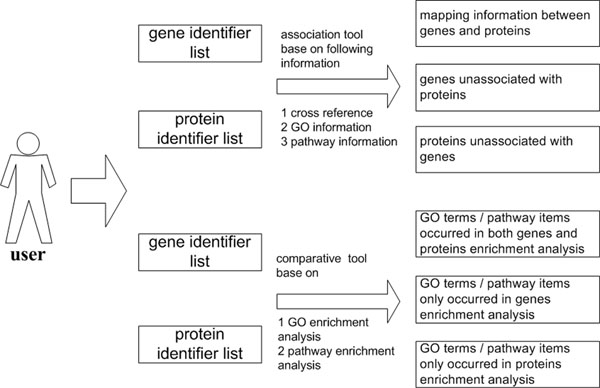
Data flow of the GePro module

Commonly, biologists want to know information of gene expression and protein translation at the same time for an interested physiological state. Therefore, combining investigation results of gene and protein function analysis is important for this purpose. After building the Gene and Protein modules, we set up the GePro module in order to present some useful combining tools. Currently, following tools are contained in this module: (1) An association tool based on cross reference indexes, which is a mapping tool for genes and proteins. In this tool, cross reference information between genes and proteins are first collected from National Center for Biotechnology Information (NCBI) and European Bioinformatics Institute (EBI). Then cross information is transformed to indexes and deposited in the MySQL database. When a gene list and a protein list are submitted to the server, cross reference indexes of the two lists are obtained from the database. Subsequently, the tool generates mappings between genes and proteins according to the cross reference indexes. (2) An association tool based on GO information, which is a mapping tool based on GO annotation. In this tool, it first receives a gene list and a protein list, and then carries out GO annotation on the two lists. Whereafter, the tool establishes connection between genes and proteins according to GO annotation results. In practice, a gene and a protein are connected when the former and the latter have identical GO terms. (3) An association tool based on pathway information, similar to association tool based on GO information. This tool builds connection between genes and proteins according to KEGG pathway annotation results. (4) A comparative analysis tool based on GO enrichment assay. This tool is designed to compare transcriptomics and proteomics data. In this tool, it receives a gene list and a protein list as the starting materials, and then GO enrichment analysis is executed on the two lists. Finally, GO terms occurred in both genes’ and proteins’ enrichment assays are feeded back. Meanwhile, GO terms occurred only in genes’ or proteins’ assays are presented as well. (5) A comparative analysis tool based on pathway enrichment assay. This tool was also designed to compare transcriptomics and proteomics data according to results of pathway enrichment assay. Data flow of the GePro module was shown in Figure 4.

### Case study

To demonstrate the efficiency of the iGepros server, we collected data in transcription and translation level from a published article [18], in which Hartl and his colleagues investigated the mouse brain transcriptome and proteome of embryonic days 9.5, 11.5, and 13.5. Differentially expressed genes and proteins between days 9.5 and 11.5 were collected for this case study, which contained 35 genes and 52 proteins [see additional file 1].

First, we used the Gene module to annotate the 35 genes, where the GO enrichment analysis tool was utilized for getting GO terms genes concentrated on. Results of GO enrichment analysis were listed in additional file 2. We found these genes were mainly associated with basic metabolic process and nervous system development according to results of GO enrichment analysis. This includes the reporting result of the published article.

Then, we used the Protein module to annotate the 52 proteins, where the protein annotation tool for KEGG pathway was utilized to get pathways proteins involved. Pathway information was summarized in additional file 3. These proteins mainly participated in basic metabolic pathways and associated with some neuro-disorder, such as Alzheimer, Parkinson, and Huntington disease. This was consistent with result of GO enrichment analysis for genes and the result of the published article.

Finally, we compared data of transcriptome and proteome through employing the GePro module, which could help us understand these genes and proteins better. On one hand, these genes and proteins were connected with the cross reference indexes. Connection result was shown in table 1. On the other hand, GO terms and pathway items genes and proteins associated together were presented through comparative analysis tool based on GO and pathway enrichment assay respectively. Common GO terms and pathways for genes and proteins were depicted in table 2 and table 3. With assistant of the GePro module, we could further survey biologic characters of these genes and proteins, which offered significant supplement to the work of published articles.

**Table 1:** Connection between the 35 genes and the 52 proteins

gene identifier	protein_identifier	gene_id	gene_name
Cirbp	P60824	12696	Cirbp
Dpysl2	O08553	12934	Dpysl2
Dpysl3	Q62188	22240	Dpysl3
Eef1b2	O70251	55949	Eef1b2
Lgals1	P16045	16852	Lgals1
Nmral1	Q8K2T1	67824	Nmral1
Rpsa	P14206	16785	Rpsa
Vim	P20152	22352	Vim

**Table 2:** GO terms of genes and proteins associated together

GO_id	gene_P_value	pro_P_value	GO_type	GO_term
GO:0045103	0.043089667	0.000809944	BP	Intermediate
GO:0006808	0.005984301	0.005778344	BP	Regulation
GO:0007399	0.017964662	0.015536482	BP	Nervous
GO:0045445	0.031519101	0.030447921	BP	Myoblast
GO:0055114	0.036032198	0.032136364	BP	Oxidation
GO:0007157	0.039247721	0.037919064	BP	Heterophilic
GO:0048856	0.049393286	0.040771834	BP	Anatomical
GO:0005534	0.002065316	0.001807151	MF	Galactose
GO:0016936	0.004126499	0.003611153	MF	Galactoside
GO:0003857	0.010285333	0.009004315	MF	3-hydroxyacyl-CoA
GO:0043236	0.012330066	0.010795772	MF	Laminin
GO:0005516	0.020122338	0.015608765	MF	Calmodulin
GO:0016614	0.001647056	0.017906341	MF	Oxidoreductase
GO:0005737	1.52E-06	7.17E-05	CC	Cytoplasm
GO:0005622	0.000132014	0.00020011	CC	Intracellular
GO:0045098	0.001877817	0.001564847	CC	Type
GO:0005853	2.04E-05	0.006245297	CC	Eukaryotic
GO:0043025	0.000513822	0.007576568	CC	Cell
GO:0015935	0.044138741	0.036914276	CC	Small
GO:0042995	0.007578928	0.042066324	CC	Cell

**Table 3:** Pathway information of genes and proteins associated together

gene identifier	protein identifier	KEGG_id	KEGG_term
Dpysl2	O08553	4360	Axon guidance
Fabp7	P11404	3320	PPAR signaling pathway
Marcks	P26645	4666	Fc gamma R-mediated phagocytosis

## Discussion

In the post-genomic era, genomics and proteomics data generated from high-throughput technologies are accumulated quickly. The biology community needs powerful tools to analyze large size gene and protein sets conveniently. Moreover, researchers want to understand biologic meaning of interested genes and proteins. Software that provides biologists with large-scale genes and proteins annotation capabilities is in a high demand. To meet this trend, we have established a web server called iGepros. Especially, the server is user-friendly and some demos are offered on-line, which make it suitable for a wide range of users even those with little or no programming experience. In addition, the iGepros server provides operations to connect genes and proteins, which allows users to compare different omics data sets. As demonstrated in the case study, users can first annotate genes and proteins with GO terms and pathway information through the Gene and Protein module. This primary annotation helps researchers decipher interested genes and proteins detected by high-throughput technology. Then, users can perform comparative analysis between transcriptomics and proteomics data by integrating genes and proteins through the GePro module, which present researchers ability to associate information in transcription and translation levels and comprehend biological nature of the investigated issue.

Currently, the iGepros server supports following model organisms: Human, Mouse, Rat, Chick, Cow, Zebrafish, Arabidopsis. In the future, more model organisms will be supported by the server. In addition, more tools for the GePro module will be developed. The server will have stronger capability for comparison of transcriptomics and proteomics data.

## Conclusions

iGepros is an integrated gene and protein analysis server, which has a powerful capability for gene and protein functional annotation as well as transcriptomics and proteomics data comparison. This on-line server allows (1) retrieval of gene informations of a model organism, (2) GO term annotation for a large set of genes or proteins, (3) pathway annotation for a large set of genes or proteins, (4) GO enrichment analysis for huge size gene and protein sets, (5) pathway enrichment analysis for huge size gene and protein sets, (6) *de novo* annotating gene and protein sequence without functional information, (7) mapping genes and proteins with cross reference information, (8) connecting genes and proteins through GO terms, (9) connecting genes and proteins through pathway information, (10) comparative analysis of transcriptomics and proteomics data on GO aspect, and (11) comparative analysis of transcriptomics and proteomics data on pathway aspect.

## Competing interests

The author(s) declare that they have no competing interests.

## Authors’ contributions

GYZ developed the server software and drafted the manuscript. GYZ and HBW carried out the case study. CCW and YXL directed the research project and revised the manuscript. All authors have read and approved the manuscript.

## Supplementary Material

Additional file 1 **Names of differentially expressed genes and proteins** Names of 35 genes and 52 proteins used in case study Click here for file

Additional file 2 **Results of GO enrichment analysis for 35 genes** Results of GO enrichment analysis in biological process, molecular function, and cellular component level Click here for file

Additional file 3 **Results of KEGG pathway annotation for 52 proteins** Pathway items proteins involved. Click here for file
