# Supplementary material for: iGepros: an integrated gene and protein annotation server for biological nature exploration
Source: BMC Bioinformatics. 2011 Dec 14;12(Suppl 14):S6. doi: 10.1186/1471-2105-12-S14-S6 (PMC3287471; doi:10.1186/1471-2105-12-S14-S6)
Supplement: Additional file 1 — Names of differentially expressed genes and proteins Names of 35 genes and 52 proteins used in case study [file 1471-2105-12-S14-S6-S1.pdf]

Supplement material

(1) Names of 35 differentially expressed genes

Hnrpf,Hspd1,Psmd4,Stmn1,Aldoa,Cirbp,Cnn3,Crmp1,Dpysl2,Dpysl3,Eef1b2,Eef1d,Fabp7,Hspa4,Ina,Ldha,Lgals1,Mif,Mtpn,Myl6,Pa2g4,Pafah1b2,Rpsa,Sh3bgrl,Vim,Cotl1,Hnrpa3,Hsd17b10,Hyou1,Marcks,Nmral1,Phgdh,Ptbp1,Ssbp1,Ubqln1

(2) Swiss-Prot AC number of 52 differentially expressed proteins

P46660,Q6NY00,Q00623,Q9DCX2,Q9BWA5,P60824,Q9DAW9,Q544F6,P31786,Q99LD8,O08553,O08553,Q62188,O70251,Q80T06,P57759,P11404,Q5NDA4,Q61425,Q3TCY3,P06467,O88569,O88569,Q9Z2X1,Q61316,Q5NCS5,Q8C2C7,Q8C2C7,Q9D6R2,P16045,P26645,Q642K0,Q8K2T1,Q8K2T1,Q9DCG9,Q9CQF3,Q6PKE6,Q9CQ60,P17742,Q61171,Q8K144,P14206,Q9ERB0,Q545B6,Q545B6,Q545B6,Q545B6,P63028,P61082,Q9R0P9,Q9R0P9,P20152
