# Supplementary material for: iGepros: an integrated gene and protein annotation server for biological nature exploration
Source: BMC Bioinformatics. 2011 Dec 14;12(Suppl 14):S6. doi: 10.1186/1471-2105-12-S14-S6 (PMC3287471; doi:10.1186/1471-2105-12-S14-S6)
Supplement: Additional file 2 — Results of GO enrichment analysis for 35 genes Results of GO enrichment analysis in biological process, molecular function, and cellular component level [file 1471-2105-12-S14-S6-S2.pdf]

Table 1 Result of GO enrichment analysis in biological process level for 35 genes

NO.	GOBPID	Pvalue	Term
1	GO:0032727	0.00199862	positive regulation of interferon-alpha production
2	GO:0031115	0.00199862	negative regulation of microtubule polymerization
3	GO:0006414	0.0039434	translational elongation
4	GO:0007052	0.00399339	mitotic spindle organization
5	GO:0006007	0.00480735	glucose catabolic process
6	GO:0030049	0.0059843	muscle filament sliding
7	GO:0006808	0.0059843	regulation of nitrogen utilization
8	GO:0006564	0.0059843	L-serine biosynthetic process
9	GO:0006566	0.0059843	threonine metabolic process
10	GO:0019642	0.0059843	anaerobic glycolysis
11	GO:0046365	0.00656128	monosaccharide catabolic process
12	GO:0009448	0.00797137	gamma-aminobutyric acid metabolic process
13	GO:0070252	0.00797137	actin-mediated cell contraction
14	GO:0019530	0.00797137	taurine metabolic process
15	GO:0046459	0.01193402	short-chain fatty acid metabolic process
16	GO:0032479	0.01193402	regulation of type I interferon production
17	GO:0007519	0.01385561	skeletal muscle tissue development
18	GO:0060134	0.0139096	prepulse inhibition
19	GO:0070584	0.01588138	mitochondrion morphogenesis
20	GO:0007399	0.01796466	nervous system development
21	GO:0006544	0.01981351	glycine metabolic process
22	GO:0021846	0.02373047	cell proliferation in forebrain
23	GO:0007019	0.02568328	microtubule depolymerization
24	GO:0034470	0.02761907	ncRNA processing
25	GO:0019318	0.02801296	hexose metabolic process
26	GO:0030330	0.02957759	DNA damage response, signal transduction by p53 class mediator
27	GO:0031110	0.02957759	regulation of microtubule polymerization or depolymerization
28	GO:0032729	0.0315191	positive regulation of interferon-gamma production
29	GO:0030048	0.0315191	actin filament-based movement
30	GO:0021782	0.0315191	glial cell development
31	GO:0045445	0.0315191	myoblast differentiation
32	GO:0006541	0.03539088	glutamine metabolic process
33	GO:0055114	0.0360322	oxidation reduction
34	GO:0060537	0.0376829	muscle tissue development
35	GO:0007157	0.03924772	heterophilic cell adhesion
36	GO:0045103	0.04308967	intermediate filament-based process
37	GO:0007569	0.04691678	cell aging
38	GO:0034660	0.04698237	ncRNA metabolic process
39	GO:0048856	0.04939329	anatomical structure development

Table 2 Result of GO enrichment analysis in molecular function level for 35 genes

NO.	GOMFID	Pvalue	Term
1	GO:0016614	0.00164706	oxidoreductase activity, acting on CH-OH group of donors
2	GO:0003746	0.00197193	translation elongation factor activity
3	GO:0004617	0.00206532	phosphoglycerate dehydrogenase activity
4	GO:0050178	0.00206532	phenylpyruvate tautomerase activity
5	GO:0005534	0.00206532	galactose binding
6	GO:0047015	0.00206532	3-hydroxy-2-methylbutyryl-CoA dehydrogenase activity
7	GO:0004167	0.0041265	dopachrome isomerase activity
8	GO:0016936	0.0041265	galactoside binding
9	GO:0019215	0.00618356	intermediate filament binding
10	GO:0016286	0.00618356	small conductance calcium-activated potassium channel activity
11	GO:0004459	0.0082365	L-lactate dehydrogenase activity
12	GO:0004332	0.01028533	fructose-bisphosphate aldolase activity
13	GO:0008307	0.01028533	structural constituent of muscle
14	GO:0003857	0.01028533	3-hydroxyacyl-CoA dehydrogenase activity
15	GO:0003847	0.01028533	1-alkyl-2-acetylglycerophosphocholine esterase activity
16	GO:0001530	0.01028533	lipopolysaccharide binding
17	GO:0043236	0.01233007	laminin binding
18	GO:0005516	0.02012234	calmodulin binding
19	GO:0015035	0.02046815	protein disulfide oxidoreductase activity
20	GO:0045182	0.02156682	translation regulator activity
21	GO:0022839	0.03054944	ion gated channel activity
22	GO:0005227	0.03054944	calcium activated cation channel activity
23	GO:0003697	0.04844304	single-stranded DNA binding

Table 3 Result of GO enrichment analysis in cellular component level for 35 genes

NO.	GOCCID	Pvalue	Term
1	GO:0005737	1.52E-06	cytoplasm
2	GO:0005853	2.04E-05	eukaryotic translation elongation factor 1 complex
3	GO:0005622	0.00013201	intracellular
4	GO:0043025	0.00051382	cell soma
5	GO:0045098	0.00187782	type III intermediate filament
6	GO:0019861	0.00379495	flagellum
7	GO:0030425	0.00545754	dendrite
8	GO:0016461	0.00562323	unconventional myosin complex
9	GO:0042995	0.00757893	cell projection
10	GO:0005829	0.02734026	cytosol
11	GO:0015935	0.04413874	small ribosomal subunit
