# Supplementary material for: iGepros: an integrated gene and protein annotation server for biological nature exploration
Source: BMC Bioinformatics. 2011 Dec 14;12(Suppl 14):S6. doi: 10.1186/1471-2105-12-S14-S6 (PMC3287471; doi:10.1186/1471-2105-12-S14-S6)
Supplement: Additional file 3 — Results of KEGG pathway annotation for 52 proteins Pathway items proteins involved. [file 1471-2105-12-S14-S6-S3.pdf]

Table 4 Pathway information of 52 proteins

protein_id	entrez_gene_id	entrez_gene_name	kegg_path_id	kegg_path_name
P46660	226180	Ina	null	null
Q6NY00	null	null	null	null
Q00623	11806	Apoa1	null	null
Q9DCX2	71679	Atp5h	190	Oxidative phosphorylation
Q9DCX2	71679	Atp5h	1100	Metabolic pathways
Q9DCX2	71679	Atp5h	5010	Alzheimer's disease
Q9DCX2	71679	Atp5h	5012	Parkinson's disease
Q9DCX2	71679	Atp5h	5016	Huntington's disease
Q9BWA5	null	null	null	null
P60824	12696	Cirbp	null	null
Q9DAW9	71994	Cnn3	null	null
Q544F6	null	null	null	null
P31786	13167	Dbi	null	null
Q99LD8	51793	Ddah2	null	null
O08553	12934	Dpysl2	4360	Axon guidance
O08553	12934	Dpysl2	4360	Axon guidance
Q62188	22240	Dpysl3	null	null
O70251	55949	Eef1b2	null	null
Q80T06	null	null	null	null
P57759	67397	Erp29	null	null
P11404	14077	Fabp3	3320	PPAR signaling pathway
Q5NDA4	null	null	null	null
Q61425	15107	Hadh	62	Fatty acid elongation in mitochondria
Q61425	15107	Hadh	71	Fatty acid metabolism
Q61425	15107	Hadh	280	Valine, leucine and isoleucine degradation
Q61425	15107	Hadh	310	Lysine degradation
Q61425	15107	Hadh	380	Tryptophan metabolism
Q61425	15107	Hadh	650	Butanoate metabolism
Q61425	15107	Hadh	1100	Metabolic pathways
Q3TCY3	null	null	null	null
P06467	15126	Hba-x	null	null
O88569	53379	Hnrnpa2b1	null	null
O88569	53379	Hnrnpa2b1	null	null
Q9Z2X1	98758	Hnrnpf	null	null
Q61316	15525	Hspa4	null	null
Q5NCS5	null	null	null	null
Q8C2C7	null	null	null	null
Q8C2C7	null	null	null	null
Q9D6R2	67834	Idh3a	20	Citrate cycle (TCA cycle)
Q9D6R2	67834	Idh3a	1100	Metabolic pathways
P16045	16852	Lgals1	null	null
P26645	17118	Marcks	4666	Fc gamma R-mediated phagocytosis
Q642K0	null	null	null	null
Q8K2T1	67824	Nmral1	null	null
Q8K2T1	67824	Nmral1	null	null
Q9DCG9	627985	Gm13072	null	null
Q9CQF3	68219	Nudt21	null	null

Q6PKE6	null	null	null	null
Q9CQ60	66171	Pgls	30	Pentose phosphate pathway
Q9CQ60	66171	Pgls	1100	Metabolic pathways
P17742	null	null	null	null
Q61171	21672	Prdx2	null	null
Q8K144	null	null	null	null
P14206	16785	Rpsa	null	null
Q9ERB0	67474	Snap29	null	null
Q545B6	null	null	null	null
Q545B6	null	null	null	null
Q545B6	null	null	null	null
Q545B6	null	null	null	null
P63028	null	null	null	null
P61082	22192	Ube2m	null	null
Q9R0P9	22223	Uchl1	null	null
Q9R0P9	22223	Uchl1	null	null
P20152	22352	Vim	null	null
